# Greigite Fe_3_S_4_ as a new anode material for high-performance sodium-ion batteries†
†Electronic supplementary information (ESI) available. See DOI: 10.1039/c6sc02716d



**DOI:** 10.1039/c6sc02716d

**Published:** 2016-08-01

**Authors:** Qidong Li, Qiulong Wei, Wenbin Zuo, Lei Huang, Wen Luo, Qinyou An, Vasiliy O. Pelenovich, Liqiang Mai, Qingjie Zhang

**Affiliations:** a State Key Laboratory of Advanced Technology for Materials Synthesis and Processing , Wuhan University of Technology , Wuhan , 430070 , China . Email: mlq518@whut.edu.cn ; Email: zhangqj@whut.edu.cn; b School of Physics and Technology , Wuhan University , Wuhan 430070 , China

## Abstract

Transition metal dichalcogenide materials have been considered as promising anode materials for rechargeable sodium-ion batteries because of their high specific capacity and low cost. Here, we demonstrate an iron sulfide Fe_3_S_4_ as a new anode material for a rechargeable sodium-ion battery. The involved conversion mechanism has been proved when the as-prepared Fe_3_S_4_ was used as the host material for sodium storage. Remarkably, a compound FeS_*x*_ with quantum size generated by conversion reaction overcame the kinetic and thermodynamic constraints of chemical conversion to achieve superior cycling and rate capability. As a result, the as-prepared Fe_3_S_4_ electrode delivers a high reversible specific capacity of 548 mA h g^–1^ at 0.2 A g^–1^, together with an excellent cycling stability of 275 mA h g^–1^ after 3500 cycles at 20 A g^–1^.

## Abstract 2

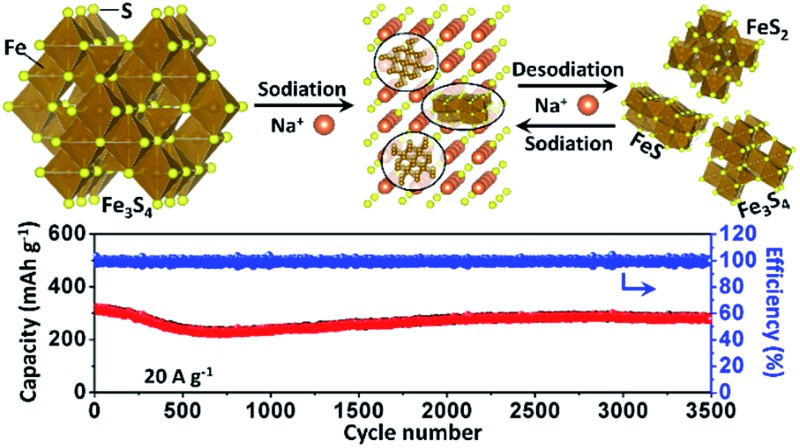
A new anode material, Fe_3_S_4_, shows superior electrochemical performance and a novel mechanism for sodium storage.

## Introduction

Owing to the increasing demand for sustainable and renewable power sources, much effort has been devoted to energy storage innovation over the past decades. Due to their high energy density and fast recharge capability, lithium-ion batteries (LIBs) have been successfully applied in many aspects of our daily life.^1,2^ In particular, the application of LIBs in electric vehicles (EVs) and hybrid EVs has reduced significantly our energy dependence on ‘one-off’ resources. Nevertheless, concerns about LIBs have arisen both in terms of their cost and the supply limits of lithium resources in recent years.^3^ Alternatively, sodium-ion batteries (SIBs) have recently attracted considerable interest owing to the low cost, wide distribution and abundant resource of sodium.^4,5^ However, compared to Li^+^ ion, the larger ionic radius and molar mass of Na^+^ ion often lead to inferior cyclability and lower specific capacity.^6,7^ There are still many challenges to exploit host materials for sodium with high capacity, fast charge–discharge, and long cycle life, especially for anode materials.^6^

The emerging transition metal dichalcogenides (TMD) materials which have been researched in electrochemistry^8–12^ for many years have drawn extensive attention for SIBs in recent years.^13–17^ These TMD materials often involve a multi-step reaction mechanism (intercalation and conversion, such as MoS_2_) which contributes a high specific capacity but with poor cycling life.^18^ Among these TMD materials, iron sulfides (FeS,^13^ FeS_2_ (ref. 15, 19 and 20)) have been researched in LIBs and SIBs numerous times owing to their high capacity, low cost and environmental friendliness. Unfortunately, the limited cycling life of iron sulfides severely restricts their real application in energy storage.^21,22^ Wang *et al.* constructed the multi-functional yolk–shell FeS@C structure to improve the cycling stability, but which could only prolong the cycling life to 300 cycles.^13^ Through controlling the cut-off voltage to avoid the conversion reaction, Hu *et al.* have improved the cycling life of iron sulfides to a quite high level (20 000 cycles) but with inferior capacity.^15^ The key point to achieve high capacity and stability simultaneously is to sustain the high reversibility of the conversion reactions. Ultrafine nanoparticles have proved to be advantageous in this respect, which is attributed to nanoparticles having a size comparable to the diffusion length of the cation in the host-materials, leading to highly reversible and efficient conversion reaction.^14^ However, it still remains a challenge to make common materials to reach the quantum size.^23^

Greigite Fe_3_S_4_, an important semi-metallic magnetic material, has been widely used in paleomagnetism, electrochemistry, biomedicine and environmental magnetic studies.^24,25^ However, to the best of our knowledge, there is no report on Fe_3_S_4_ as the anode of SIBs. Herein, we demonstrate Fe_3_S_4_ as a promising host-material for sodium storage. The involved conversion reaction pulverizes the Fe_3_S_4_ particles to quantum size during the sodiation/desodiation processes, resulting in a high capacity and superior stability. The synthesized Fe_3_S_4_ particles display a discharge capacity of 548 mA h g^–1^ in a wide operating voltage between 0.5 and 3 V. Meanwhile, the remarkable long-term cyclic stability (275 mA h g^–1^ after 3500 cycles at 20 A g^–1^) and excellent rate capability (233 mA h g^–1^ at 40 A g^–1^) assure its great potential for practical utilization. This high reversible conversion mechanism presents a new method to enable SIBs possessing both high capacity and long-cycle stability.

## Results and discussion

**Fig. 1:**
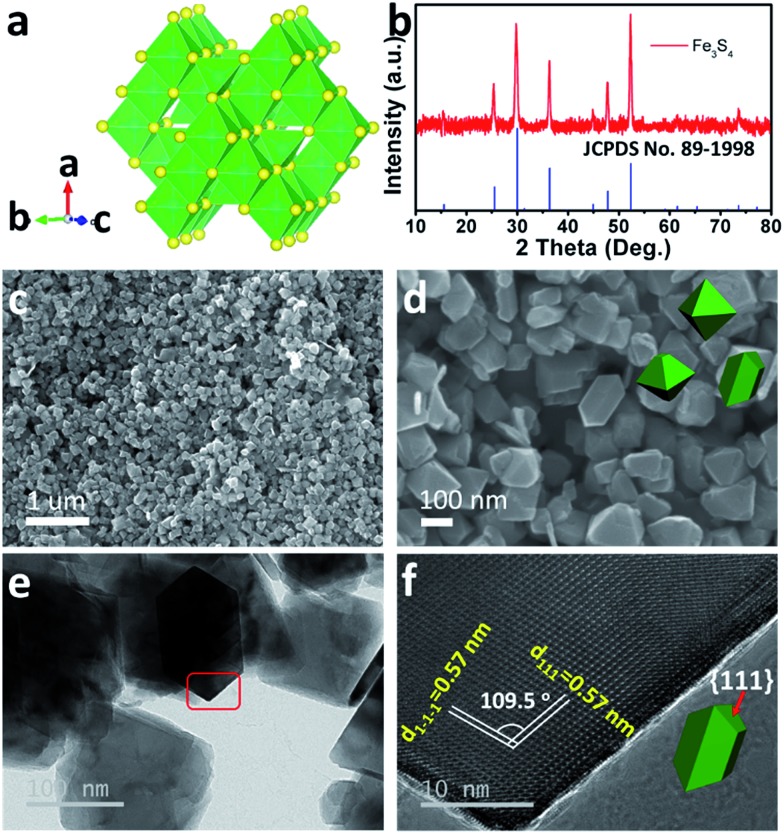
Structure characterization of the as-prepared Fe_3_S_4_. (a) Crystal structure and (b) XRD pattern of the as-prepared Fe_3_S_4_. (c and d) SEM images, (e) TEM and (f) HRTEM images for the as-prepared Fe_3_S_4_.

As the counterpart of the oxide magnetite Fe_3_O_4_, greigite Fe_3_S_4_ contains 32 atoms of sulfur and 24 atoms of iron per unit cell. There are two sublattices of iron atoms where the Fe^3+^ ions occupy tetrahedral A-sites and both Fe^2+^ and Fe^3+^ ions occupy octahedral B-sites (Fig. 1a).^25^ Fig. 1b shows the X-ray diffraction (XRD) pattern of the as-prepared Fe_3_S_4_. All diffraction peaks are fully consistent with JCPDS no. 89-1998, showing a cubic *Fd*3*m* space group. The morphology of the as-prepared Fe_3_S_4_ is characterized by scanning electron microscopy (SEM) (Fig. 1c and d). The particles present octahedral features and the particle size is 100–200 nm. Fig. 1f clearly shows two lattices: (111) and (1–1–1), which are parallel to the surface of the octahedron. The intersection angle of these two lattice plane is measured to be 109.5°, which is consistent with the theoretical value. Combining the crystal structure of Fe_3_S_4_ (Fig. 1a), it is speculated that the exposed faces are the {111} family of crystal planes.

**Fig. 2:**
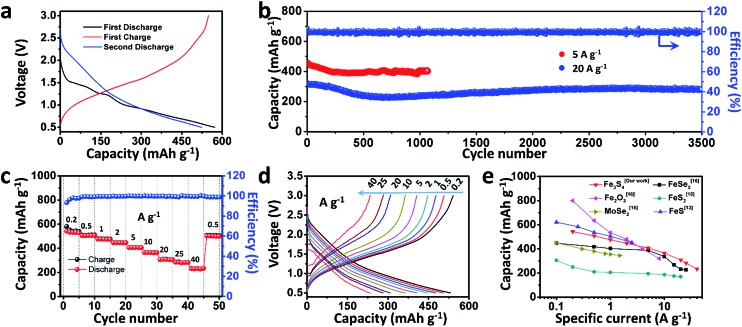
Electrochemical performance of the as-prepared Fe_3_S_4_. (a) Discharge–charge curves of the first two cycles at a specific current of 0.2 A g^–1^; (b) cycling performances at 5 and 20 A g^–1^; (c) rate capability and (d) discharge–charge curves at various current rates; (e) comparison of the as-prepared Fe_3_S_4_ with other conversion anode materials of SIBs.

To test the electrochemical performances of the as-prepared Fe_3_S_4_ particles, CR2016 coin-type cells were fabricated. Fig. 2a shows the galvanostatic discharge/charge processes for the as-prepared Fe_3_S_4_ electrode at a low current rate of 0.2 A g^–1^ in a range of 0.5–3.0 V. The Fe_3_S_4_ delivers a high initial discharge capacity of 571 mA h g^–1^, and the first charge capacity is 548 mA h g^–1^, which shows an impressive initial coulombic efficiency of 96%. After 100 cycles, it still delivers a reversible discharge capacity of 536 mA h g^–1^ (Fig. S1†). The cyclic voltammogram (CV) curves of the as-prepared Fe_3_S_4_ electrode at a scan rate of 0.2 mV s^–1^ show that the charge and discharge processes maintain stable curves after the initial cycle (Fig. S2a†). The long-term cycling is tested under a relatively high specific current (5 and 20 A g^–1^) as shown in Fig. 2b and S3.† After 50 cycles, the Fe_3_S_4_ anode delivers a stable discharge capacity of 435 mA h g^–1^ at 5 A g^–1^. After 1000 cycles, a capacity of 401 mA h g^–1^ is still obtained, showing an impressive cycling stability. Moreover, the shape of the capacity–voltage curves shows little change during cycling especially after 200 cycles, which confirms the stable and reversible discharge/charge processes (Fig. S2b†). The coulombic efficiency is kept at nearly 100% from beginning to end at such a high specific current. More attractively, a relatively high capacity of 275 mA h g^–1^ is also obtained after 3500 cycles even at a high specific current of 20 A g^–1^ (Fig. 2b). It should be pointed out that the superior cycling performance benefits from both the ether based electrolyte and the cut-off voltage, as shown in (Fig. S4†). Carbonate-based electrolytes (NaClO_4_/EC-DMC) suffer from rapid capacity fading (584 mA h g^–1^ at the first cycle and 15 mA h g^–1^ at the 200th cycle respectively, Fig. S4a and b†). Moreover, when we extend the operating voltage to 0.01–3 V, the capacity seriously fades from 748 to 132 mA h g^–1^ within 100 cycles at 2 A g^–1^ (Fig. S4c and d†).

The rate capabilities of the as-prepared Fe_3_S_4_ electrode are further investigated at various specific currents ranging from 0.2 to 40 A g^–1^ (Fig. 2c). Fig. 2d exhibits the corresponding charge and discharge curves of the Fe_3_S_4_ electrode at different specific currents. The capacities show slight decline as the specific current gradually increases. When the specific currents reach 0.2, 0.5, 1, 2, 5, 10, 20, and 25 A g^–1^, the discharge capacities are 548, 508, 476, 446, 407, 365, 308, and 283 mA h g^–1^, respectively. It is noteworthy that even at an extremely high specific current of 40 A g^–1^, a capacity of 233 mA h g^–1^ is still achieved, corresponding to 43% capacity utilization within 21 s. Its corresponding areal current densities and areal capacities are shown in Table S1.† Comparing the excellent rate performance with the state of the art conversion type anode materials, the Fe_3_S_4_ anode has a distinct advantage at high specific currents (Fig. 2e).^13,15,16^

### Sodium-storage mechanism

**Fig. 3:**
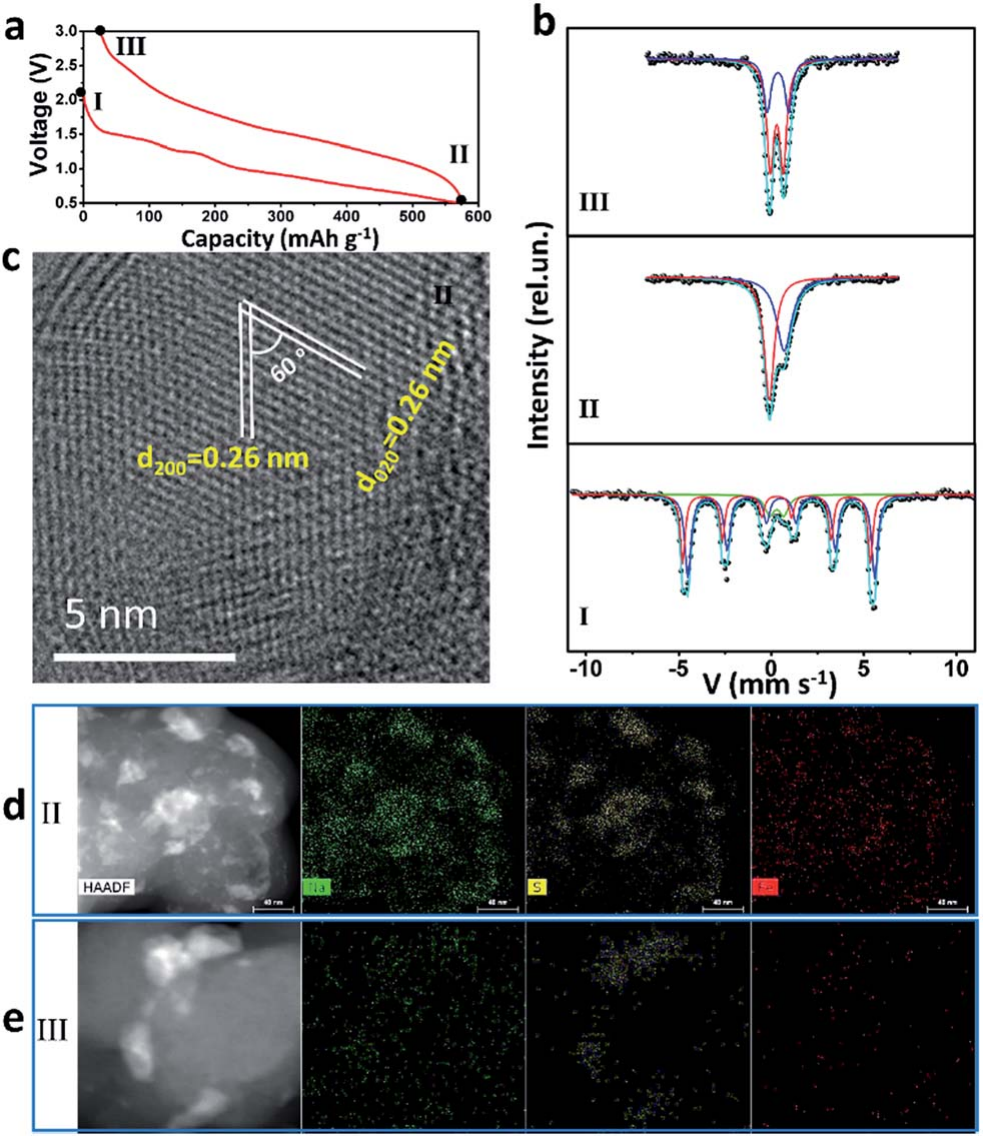
(a) Discharge–charge curves of the first cycle at the specific current of 0.2 A g^–1^; (b) ^57^Fe Mössbauer spectra, (c) TEM image, (d) STEM and (e) EDS mappings of the as-prepared Fe_3_S_4_ at different states.

^57^Fe Mössbauer spectra and TEM were used to study intensively the sodium-storage mechanism of the as-prepared Fe_3_S_4_ particles (Fig. 3). Three states have been chosen to investigate the mechanism. The first charge and discharge curves (I (fresh state) → II (discharge to 0.5 V) → III (charge to 3 V)) of the Fe_3_S_4_-based battery at a specific current of 0.2 A g^–1^ are shown in Fig. 3a. The sodium-storage mechanism is first revealed *via* ^57^Fe Mössbauer spectra. The representative ^57^Fe Mössbauer spectra recorded at room temperature for all samples are shown in Fig. 3c. The pristine Fe_3_S_4_ sample consists of two magnetic sextets and one central doublet, shown in Table S1.† Two sextets represent hyperfine interactions of Fe ions in octahedral and tetrahedral sites. The quadrupole doublet is probably associated with thermally relaxed fine particles present in the sample but already not visible by XRD.^26^ The obtained data is in good agreement with the previous findings for natural and synthetic greigite.^27^ The spectra of discharged and charged samples represent no magnetic sextets due to superparamagnetic behavior of small particles. The center shift (CS), quadrupole splitting (QS), and area ratio (*A*) obtained from analysis of the spectra are listed in Table S1.† For the discharged sample, the observed CS value of the bigger singlet (singlet 1) is –0.08 mm s^–1^, which clearly indicates that the iron is present in the nanostructured metallic state (α-Fe), the smaller singlet (singlet 2) can be attributed to nanoparticles of hexagonal FeS.^28^ Moreover, the HRTEM image is collected at the state II (Fig. 3c), which displays two sets of parallel fringes with the same *d*-spacing of 0.26 nm and an included angle of 60° between them, corresponding to the (200) and (020) planes of FeS (JCPDF no. 37-0477), which is consistent with the results obtained by Mössbauer techniques. To verify the state of sulphur, scanning TEM (STEM) and energy dispersive X-ray spectrometer (EDS) mappings were collected at state II (Fig. 3d) and III (Fig. 3e). When discharged to 0.5 V, the distribution of Na is well consistent with that of S, verifying the formation of Na_2_S. Fe shows a uniform distribution. Therefore, the initial discharge reaction can be expressed as eqn (S1).†

According to this equation, the theoretical capacity of Fe_3_S_4_ when discharging to 0.5 V is calculated to be 543 mA h g^–1^, which is very consistent with the reversible capacity of 548 mA h g^–1^ obtained at 0.2 A g^–1^. Furthermore, the Fe^0^ produced during the conversion reaction improves the conductivity of the electrode significantly, which is confirmed by the electrochemical impedance spectrum (EIS) (Fig. S5†). The EIS spectrum shows two compressed semicircles from the high to medium frequency range of each spectrum at the state II, for which the second semicircle describes the charge transfer resistance (*R*_ct_) of the electrode. After simulation, the values of *R*_ct_ for the state I and II electrodes are calculated to be 98.8 and 10.7 Ω, respectively. The improved conductivity after the formation of Fe has great benefits for high-rate performance.^29^

For charging process, the Mössbauer spectra for the charged sample can be fitted with two doublets with similar CS values but different QS values of 0.69 and 1.15 mm s^–1^. The first more-intense doublet can be attributed to the ferrous low spin Fe^2+^ state, probably in tetrahedral FeS_*x*_.^30^ The nature of the second doublet is unclear, and the ferric ion Fe^3+^ state can be caused by some other iron sulfide species. These results indicate that, after first charging to 3 V, the active materials exist in the form of compounds, which can be defined as FeS_*x*_. The FeS_*x*_ is also confirmed by the HRTEM image at state III (Fig. S6†), which is composed of FeS_2_, Fe_3_S_4_ and FeS. Notably, due to the conversion reaction, the FeS_*x*_ compounds are pulverized to nanocrystals with the size of ∼1–10 nm (Fig. S6b†). This quantum size is of great significance for iron sulfide to achieve improved cycling and better rate capability because of the shorter diffusion lengths of Fe in iron sulfide (*L*_D_ = 10^–17^ cm^2^ s^–1^ at 100 °C or ∼10^–18^ cm^2^ s^–1^ at room temperature in FeS_2_).^14,31,32^ The compound FeS_*x*_ with quantum size, which is comparable or smaller than the Fe diffusion distance during cation exchange, overcomes the significant kinetic and thermodynamic constraints of chemical conversion to achieve an excellent cycling and rate capability.^14^ After charging back to 3 V, the elemental mapping images display the uniform distribution of Na and Fe, but the S is still concentrated in some areas (Fig. 3e). These results demonstrate that the S^2–^ is at least in part transformed into S^0^. Therefore, the possible reaction during the reversible charging processes is summarized as eqn (S2).†

**Fig. 4:**
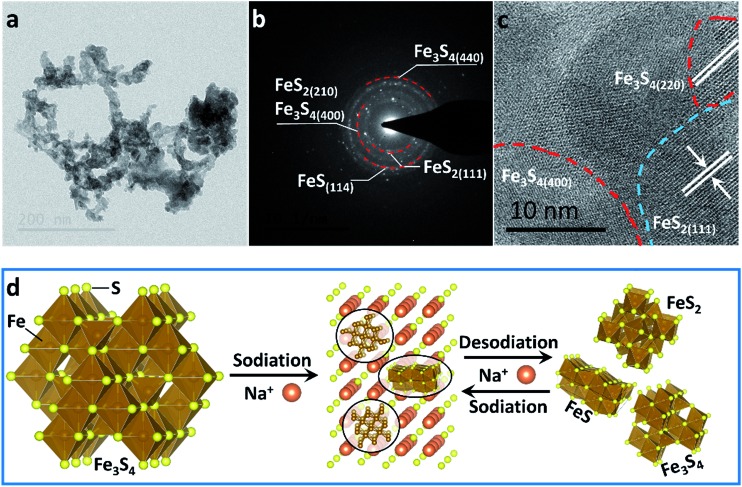
(a) TEM image, (b) SAED pattern and (c) HRTEM image of the as-prepared Fe_3_S_4_ at a full charge state after 200 cycles. (d) Schematic illustration of the sodium-storage mechanism in the Fe_3_S_4_ electrode.

To confirm the active material in the subsequent cycles, TEM images of the electrode at full charge state after 200 cycles were collected (Fig. 4). The Selected Area Electron Diffraction (SAED) patterns confirm the coexistence of Fe_3_S_4_, FeS_2_ and FeS (Fig. 4b). The HRTEM image shows the nanocrystal of Fe_3_S_4_ and FeS_2_ (Fig. 4c). The whole sodium-storage mechanism is illustrated in Fig. 4d. In the sodiation process, Na^+^ exchanges with Fe^*x*+^ to form Na_2_S, and the exchanged Fe^*x*+^ obtain electrons to form Fe^0^. A portion of Fe^*x*+^ still occupies octahedral sites to form FeS due to the controlled cut-off voltage. In the sodiation process, Fe^0^ exchanges with Na^+^ to form the Fe–S tetrahedron or octahedron, which further assembles to form the quantum-sized FeS_2_, Fe_3_S_4_ and FeS. The quantum-sized FeS_*x*_ insure a synergistic and highly reversible conversion reaction which results in the superior cyclability and rate capability.^14,33^

## Conclusions

In summary, Fe_3_S_4_ particles have been prepared successfully and used as an anode material for SIBs for the first time. A conversion mechanism with 6 Na^+^ per formula has been proved between a wide operating window of 0.5–3 V. Due to the conversion reaction, Fe_3_S_4_ particles are pulverized to the quantum-sized compound FeS_*x*_ which is composed of FeS, FeS_2_ and Fe_3_S_4_ quantum dots. The quantum-sized FeS_*x*_ insure a synergistic and high reversible conversion reaction to provide the electrode with excellent cyclability and rate capability. As a result, Fe_3_S_4_ delivers a stable discharge capacity of 275 mA h g^–1^ after 3500 cycles at 20 A g^–1^. Even at 40 A g^–1^, a high discharge capacity of 233 mA h g^–1^ is obtained. This remarkable performance makes Fe_3_S_4_ a promising application candidate for the development of SIBs with high-rate capability and long-term cyclability. We believe that this high reversible conversion mechanism provides a new direction to improve the electrochemical performance of TMD materials for SIBs. Moreover, the involved electrochemical pulverization process provides a new route to synthesise quantum-sized materials.

## References

[cit1] Kang B., Ceder G. (2009). Nature.

[cit2] Li L., Jacobs R., Gao P., Gan L., Wang F., Morgan D., Jin S. (2016). J. Am. Chem. Soc..

[cit3] Tarascon J.-M., Armand M. (2001). Nature.

[cit4] Kim S. W., Seo D. H., Ma X., Ceder G., Kang K. (2012). Adv. Energy Mater..

[cit5] Palomares V., Casas-Cabanas M., Castillo-Martínez E., Han M. H., Rojo T. (2013). Energy Environ. Sci..

[cit6] Palomares V., Serras P., Villaluenga I., Hueso K. B., Carretero-González J., Rojo T. (2012). Energy Environ. Sci..

[cit7] Islam M. S., Fisher C. A. (2014). Chem. Soc. Rev..

[cit8] Sun Y., Liu C., Grauer D. C., Yano J., Long J. R., Yang P., Chang C. J. (2013). J. Am. Chem. Soc..

[cit9] Kornienko N., Resasco J., Becknell N., Jiang C.-M., Liu Y.-S., Nie K., Sun X., Guo J., Leone S. R., Yang P. (2015). J. Am. Chem. Soc..

[cit10] Wong A. B., Brittman S., Yu Y., Dasgupta N. P., Yang P. (2015). Nano Lett..

[cit11] Xu X., Fan Z., Yu X., Ding S., Yu D., Lou X. W. D. (2014). Adv. Energy Mater..

[cit12] Cao X., Tan C., Zhang X., Zhao W., Zhang H. (2016). Adv. Mater..

[cit13] Wang Y.-X., Yang J., Chou S.-L., Liu H. K., Zhang W.-x., Zhao D., Dou S. X. (2015). Nat. Commun..

[cit14] Douglas A., Carter R., Oakes L., Share K., Cohn A. P., Pint C. L. (2015). ACS Nano.

[cit15] Hu Z., Zhu Z., Cheng F., Zhang K., Wang J., Chen C., Chen J. (2015). Energy Environ. Sci..

[cit16] Zhang K., Hu Z., Liu X., Tao Z., Chen J. (2015). Adv. Mater..

[cit17] Li W. J., Han C., Chou S. L., Wang J. Z., Li Z., Kang Y. M., Liu H. K., Dou S. X. (2016). Chem.–Eur. J..

[cit18] David L., Bhandavat R., Singh G. (2014). ACS Nano.

[cit19] Kim T., Choi J., Ryu H., Cho G., Kim K., Ahn J., Cho K., Ahn H. (2007). J. Power Sources.

[cit20] Kim T., Jung W., Ryu H., Kim K., Ahn J., Cho K., Cho G., Nam T., Ahn I., Ahn H. (2008). J. Alloys Compd..

[cit21] Fong R., Dahn J., Jones C. (1989). J. Electrochem. Soc..

[cit22] Golodnitsky D., Peled E. (1999). Electrochim. Acta.

[cit23] Gu H., Zheng R., Zhang X., Xu B. (2004). J. Am. Chem. Soc..

[cit24] He Z., Yu S. H., Zhou X., Li X., Qu J. (2006). Adv. Funct. Mater..

[cit25] Lyubutin I., Starchikov S., Lin C.-R., Lu S.-Z., Shaikh M. O., Funtov K., Dmitrieva T., Ovchinnikov S., Edelman I., Ivantsov R. (2013). J. Nanopart. Res..

[cit26] Chang L., Roberts A. P., Tang Y., Rainford B. D., Muxworthy A. R., Chen Q. (2008). J. Geophys. Res..

[cit27] Vandenberghe R., De Grave E., De Bakker P., Krs M., Hus J. (1992). Hyperfine Interact..

[cit28] Kim W., Park I. J., Kim C. S. (2009). J. Appl. Phys..

[cit29] Xie Q., Ma Y., Wang X., Zeng D., Wang L., Mai L., Peng D.-L. (2016). ACS Nano.

[cit30] Dékány I., Turi L., Homonnay Z., Vértes A., Burger K. (1996). Colloids Surf., A.

[cit31] McDowell M. T., Lu Z., Koski K. J., Yu J. H., Zheng G., Cui Y. (2015). Nano Lett..

[cit32] Chen J., Harvey W. (1975). Metall. Trans. B.

[cit33] Qian J., Xiong Y., Cao Y., Ai X., Yang H. (2014). Nano Lett..

