# Supplementary material for: Greigite Fe3S4 as a new anode material for high-performance sodium-ion batteries
Source: Chem Sci. 2016 Aug 1;8(1):160–4. doi: 10.1039/c6sc02716d (PMC5308403; doi:10.1039/c6sc02716d)
Supplement: Supplementary file 1 [file SC-008-C6SC02716D-s001.pdf]

Electronic Supplementary Material (ESI) for Chemical Science.
This journal is © The Royal Society of Chemistry 2016

Supporting Information

Greigite Fe_3S_4 as a New Anode Material for High-Performance Sodium-Ion Batteries

Qidong Li,[a]‡ Qiulong Wei,[a]‡ Wenbin Zuo,[b] Lei Huang,[a] Wen Luo,[a] Qinyou An,[a] Vasiliy O.Pelenovich,[b] Liqiang Mai*[a] and Qingjie Zhang*[a]

a State Key Laboratory of Advanced Technology for Materials Synthesis and Processing, Wuhan University of Technology, Wuhan, 430070, China.

b School of Physics and Technology, Wuhan University, Wuhan 430070, China

‡ Q. L. and Q. W. contributed equally.

*Email: mlq518@whut.edu.cn; zhangqj@whut.edu.cn.

Experimental Section

Synthesis of Fe_3S_4 particles. In a typical synthesis, 2 mmol $Fe(NO_3)_3 \cdot 9H_2O$ was dissolved in 40 mL glycerinum. Then the solution was transferred into a 50 mL Teflon-lined sealed autoclave and maintained at 140 ^oC for 12 h. The obtained precursor was washed thoroughly with absolute ethyl alcohol. Afterwards, the precursor was dispersed in 80 mL ethyl alcohol without drying. After that, 10 mmol thioacetamide (TAA) was dissolved in the suspension via magnetic stirring and ultrasonic. Then the green suspension was transferred into a 100 mL Teflon-lined sealed autoclave and maintained at 120 ^oC for 12 h. The precipitate collected by centrifugation was washed with ethyl alcohol several times and dried in a vacuum oven at 70 °C.

Material characterization. XRD patterns of the samples were obtained with a D8 Advance X-ray diffractometer, using Cu-Kα radiation (λ = 1.5418 Å). Field emission scanning electron microscopy images were collected with a JEOL-7100F microscope. TEM and HRTEM images were recorded by using a JSM-2010 microscope. Element mapping was tested by using a EDX-GENESIS 60S spectrometer. The Mössbauer spectra were collected by a conventional spectrometer in transmission geometry. As a gamma ray source 57Co in Rh matrix with an activity of 2 mCi was applied. For velocity calibration α-Fe foil as the calibration absorber was used. The Recoil code was used to fit the spectra. The peak area ratios of the sextets were set as 3:2:1:1:2:3.

Electrochemical test. The electrochemical properties were characterized in 2016 coin cells with sodium foils as the anode. The working electrodes were prepared by mixing 80 wt% active material, 15 wt% acetylene black and 5 wt% carboxyl methyl cellulose (CMC) binder. The slurry was cast onto Cu foil by using a doctor blade and dried in a vacuum oven at 70 °C for 8 h. The mass loading of active materials was 0.8–1.5 mg cm^{-2}. The electrolyte is composed of 1.0 M $NaCF_3SO_3$ dissolved in diethyleneglycol dimethylether (DEGDME). Galvanostatic charge/discharge measurements were performed using a multichannel battery testing system (LAND CT2001A). Cycling voltammetry (CV) and electrochemical impedance spectroscopy (EIS) were collected with an Autolab potentiostat/galvanostat at room temperature.

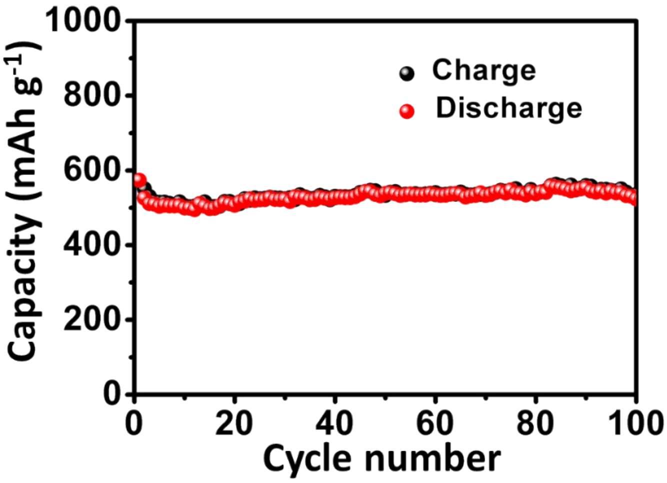

Figure S1. The cycling performance of the as-prepared Fe_3S_4 at 0.2 A g^{-1}.

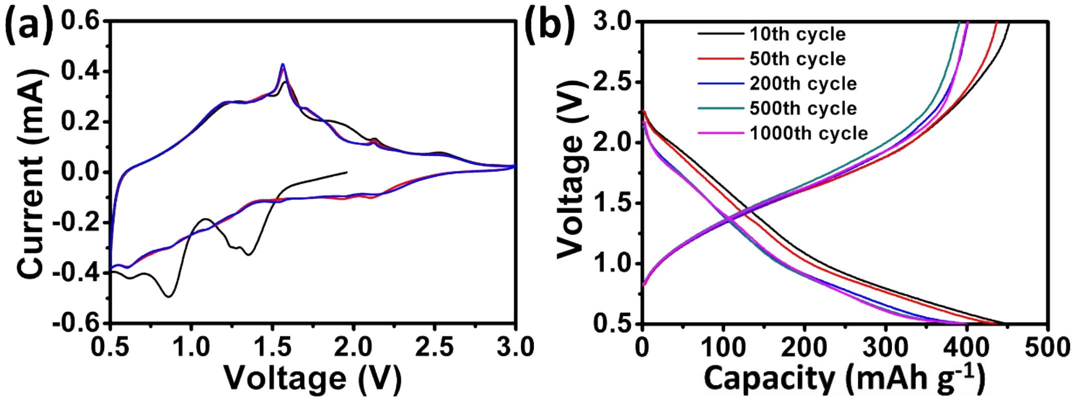

Figure S2. Electrochemical performance of the as-prepared Fe_3S_4. (a) The cyclic voltammogram (CV) curves of the first three cycles at a scan rate of 0.2 mV s^{-1}; (b) discharge–charge curves at different cycles of the as-prepared Fe_3S_4 at 5 A g^{-1}.

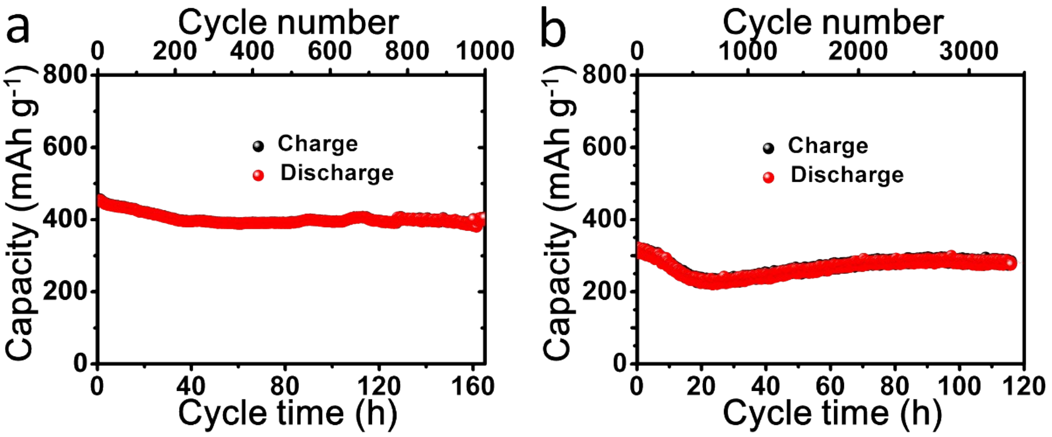

Figure S3. The corresponding cycling performances of the as-prepared Fe_3S_4 labeled by cycle time (hours) at (a) 5 A g^{-1} and (b) 20 A g^{-1}.

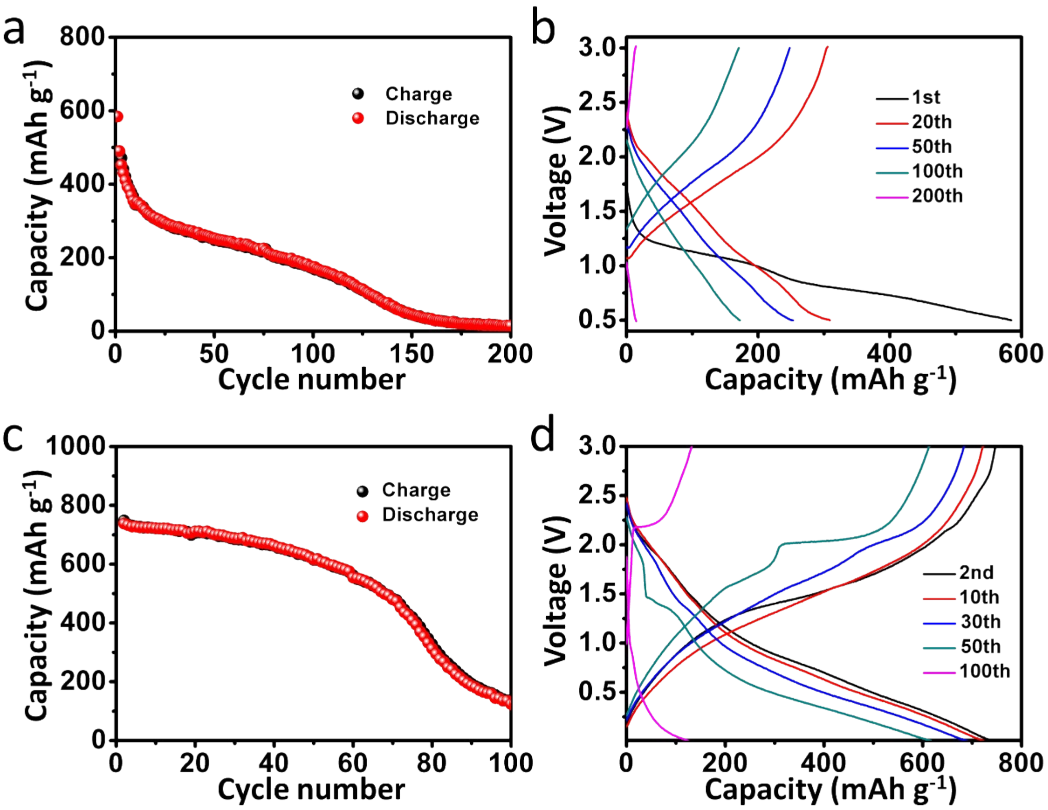

Figure S4. Electrochemical performance of the as-prepared Fe_3S_4. (a) The cycling performance at 2 A g^{-1} in $NaClO_4$/EC-DMC; (b) discharge–charge curves at different cycles of the as-prepared Fe_3S_4 at 2 A g^{-1} in $NaClO_4$/EC-DMC; (c) cycling performance at 2 A g^{-1} in NaSO3CF3/DGM between 0.01-3 V; (d) discharge–charge curves at different cycles of the as-prepared Fe_3S_4 at 2 A g^{-1} in NaSO3CF3/DGM between 0.01-3 V.

Table S1. The corresponding areal current density and areal capacity for the rate performance of the as-prepared Fe_3S_4.

Current density (mA cm^{-2})	0.24	0.6	1.2	2.4	6	12	24	30	48
Capacity (mAh cm^{-2})	0.657	0.610	0.571	0.535	0.488	0.438	0.369	0.339	0.279

Table S2. The Mössbauer parameters.

sample		CS (mm/s)	QS (mm/s)	A (%)	Iron state
pristine	Doublet 1	0.29±0.04	-	10	Fe^{3+}
	Sextet 1	0.29±0.01	-0.01±0.01	36	Tetrahedral Fe^{3+}
	Sextet 2	0.54±0.01	0.00±0.01	54	Octahedral $Fe^{2.5+}$
discharged	Singlet 1	-0.08±0.01	-	52	Fe
	Singlet 2	0.72±0.01	-	48	Fe^{2+}
charged	Doublet 1	0.32±0.01	0.69±0.04	67	Low spin Fe^{2+}
	Doublet 2	0.39±0.02	1.15±0.06	33	Fe^{3+}

The possible reaction equations of the discharge/charge process of the as-prepared Fe_3S_4.

Discharge: $Fe_3S_4 + 6Na^+ + 6e^- \rightarrow 2Fe + 3Na_2S + FeS$ (S1)

Charge: $2Fe + 3Na_2S + FeS \rightarrow 3FeS_x + (4/3 - 3x)S + 6Na^+ + 6e^-$ (S2)

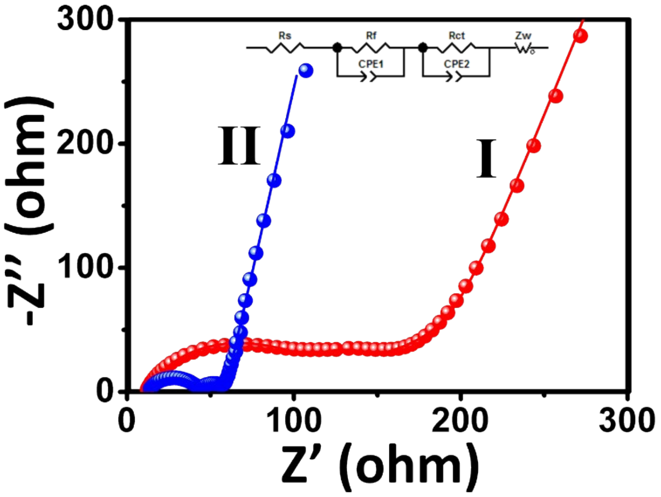

Figure S5. Electrochemical impedance spectrum (EIS) at different state.

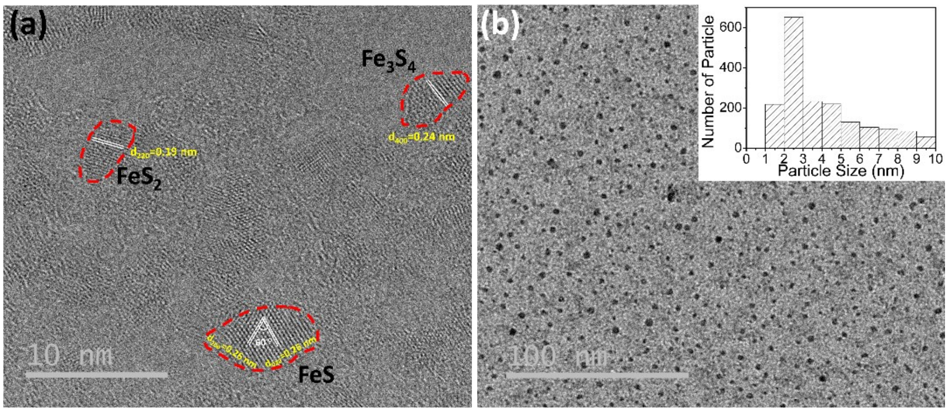

Figure S6. (a) HRTEM image, (b) TEM image and the particle size distribution (insert image) of the as-prepared Fe_3S_4 at state III.
